# The therapeutic effect of stem cells from human exfoliated deciduous teeth on a rat model of tracheal fistula

**DOI:** 10.1186/s13287-022-02994-x

**Published:** 2022-07-15

**Authors:** Fang Wang, Zhangwen Li, Feng-Juan Lyu, Jie Gao, Jinle Lin, Jianling Liu, Xiaowen Chen, Zhongpeng Li, Jiajie Shan, Jian Wu

**Affiliations:** 1 School of Medicine, South China University of Technology, Guangzhou, 510006 China; 2 Second Department of Elderly Respiratory, Guangdong Provincial People’s Hospital, Guangdong Academy of Medical Sciences, Guangdong Provincial Geriatrics Institute, Guangzhou, 510080 China; 3 The Second School of Clinical Medicine, Southern Medical University, Guangzhou, 510515 China; 4 Department of Emergency Medicine, Affiliated Baoan Hospital of Shenzhen, The second school of clinical medicine, Southern Medical University, Shenzhen, 518101 China

**Keywords:** Stem cells from human exfoliated deciduous teeth, Tracheal fistula, MSCs, Cell therapy

## Abstract

**Background:**

Tracheal fistulas (TF) can be dangerous and even fatal in patients. The current treatment is really challenging. Previous studies reported that mesenchymal stem cells (MSCs) could be used to treat respiratory tract fistulas. Stem cells from human exfoliated deciduous teeth (SHED) are considered to be MSC-like cells that may also have the potential to treat the tracheal fistulas. In this study, we investigated the therapeutic effects of SHED in rat tracheal fistula models.

**Methods:**

A total of 80 SD rats were randomly divided into five groups: a sham-operated group, a local PBS group (L-PBS), an intravenous PBS group (I-PBS), a local SHED treatment group (L-SHED), and an intravenous SHED treatment group (I-SHED). The L-SHED and I-SHED groups were given a topical application around the fistula or an intravenous injection of 1*10^7^ SHED via the tail vein, respectively, while the L-PBS and I-PBS groups were given an equivalent volume of PBS through local or intravenous administration. A stereomicroscope was used to observe fistula healing on the 2nd, 3rd, and 5th days following transplantation. On the 7th day, the survival of SHED was observed by immunofluorescence. The pathology of the lungs and fistulas was observed by hematoxylin and eosin (H&E) and Masson staining. The expression levels of the Toll-like receptor 4 (TLR4), interleukin (IL)-1β, IL-33, and IL-4 were measured using immunohistochemistry. The expression levels of TLR4, high mobility group box 1 (HMGB1), and myeloid differentiation factor 88 (MYD88) were studied using western blotting. On day 14, airway responsiveness of rats was detected and analyzed.

**Results:**

Fistula healing in the L-SHED and I-SHED groups was faster than that in their respective PBS groups after transplantation. The fistula diameters in the L-SHED and I-SHED groups were significantly smaller than those in the L-PBS and I-PBS groups on the 3rd day. Moreover, the phenomenon of fibroblast proliferation and new blood vessel growth around the fistula seemed more pronounced in the L-SHED and I-SHED groups. Although no discernible difference was found in airway responsiveness after SHED treatment, the degree of inflammation in the lungs was reduced by intravenous SHED treatment. However, there was no significant reduction in lung inflammation by local SHED treatment. The expression levels of IL-1β and IL-33 were decreased in the I-SHED group, while IL-4 was elevated compared with the I-PBS group. Interestingly, intravenous SHED treatment inhibited the activation of HMGB1/TLR4/MYD88 in the lung tissues of TF rats.

**Conclusions:**

SHED transplantation accelerated the rate of fistula healing in rats. Intravenous SHED treatment reduced lung inflammation. Thus, SHED may have potential in the treatment of tracheal fistula, providing hope for future therapeutic development for TF.

## Introduction

A respiratory tract fistula is an abnormal passage communicating between any component of the respiratory tract or between any part of the respiratory system and surrounding organs [1], including tracheal fistula (TF) and bronchial fistula (BF). Tracheal fistula includes tracheoesophageal fistula (TEF) and tracheomediastinal fistula (TMF). Bronchoesophageal fistula (BEF), bronchopleural fistula (BPF), bronchocutaneous fistula, and bronchobile duct fistula are all bronchial fistula types. Tracheal fistula occurs in cases of prolonged mechanical ventilation and excessive cuff pressure of the endotracheal tube or tracheostomy tube, blunt trauma, infections, stent-related injuries, ingestion of foreign bodies or corrosive products, and cancers arising from the esophagus, trachea, lungs, larynx, and thyroid [2–4]. Sometimes, small fistulas can close spontaneously, while fistulas larger than 20 mm rarely heal spontaneously, resulting in a low survival rate [5]. Currently, the most common treatments for TF are surgery, interventional therapy, and conservative treatments [6]. However, these therapies do have some drawbacks. Managing TF currently remains challenging, and there are no viable techniques available [7, 8]. Mesenchymal stem cells (MSCs) have recently been found to promote damaged tissue healing [9] and alleviate local inflammatory responses, giving patients a unique therapeutic alternative.

Several pioneering studies have explored the therapeutic efficacy of MSC administration for treating respiratory tract fistulas. In a goat model of BPF, Petrella et al. [10] found that bronchoscopic stem cell transplantation enhanced fistula healing by extraluminal fibroblast proliferation and collagenous matrix development. Encouraged by this result, he presented a case report of effective BPF blockage with MSC injection under bronchoscopy [11]. At 60 days, bronchoscopy showed complete healing of the resection line and the orifice that was observed before stem cell implantation was no longer visible. Current evidence supports the safety of MSCs in treating respiratory tract fistula and suggests a beneficial effect. However, research in this field is still scarce, and the molecular mechanisms remain unknown. There are no reports on animal models of tracheal fistulas and no studies on stem cell therapy of tracheal fistulas.

MSCs can be isolated from many tissues and organs [12] and have slight differences in their surface marker expression and differentiation potential [13]. Stem cells from human exfoliated deciduous teeth (SHED), first discovered in 2003 [14], are self-renewing MSCs residing within the perivascular niche of the dental pulp. They are derived from the neural crest and can differentiate into osteoblasts, chondrocytes, hepatocytes, and neuron-like cells under appropriate conditions. Because teeth fall out naturally throughout childhood, offering the ideal chance to recover and retain this easy supply of young stem cells, obtaining SHED is simple and convenient, with little or no trauma and fewer ethical concerns. Furthermore, the dental pulp of deciduous teeth is present from birth and lasts until permanent teeth erupt. This period is characterized by maintaining an active niche rich in stem cells, which are strong and proliferative and are not yet heavily affected by the cumulative effects of genetic and environmental factors [15]. SHED have been shown to promote considerable recovery in many animal models of disease, including liver fibrosis, systemic lupus erythematosus, ischemic brain injury, spinal cord injury, and diabetes [16–22].

In this study, we successfully established a simple rat tracheal fistula model. With this model, we investigated the curative effects and probable mechanisms of SHED on TF. In our study, SHED transplantation accelerated the rate of fistula healing in rats. Most importantly, intravenous SHED treatment reduced lung inflammation and inhibited the activation of the HMGB1/TLR4/MYD88 signaling pathway in the lung tissues of TF rats.

## Materials and methods

### Origin and identification of SHED

SHED at passage 4 were present from CAR-T (Shanghai) Biotechnology Co., Ltd. and were isolated from individuals (aged 6–12 years) who all signed informed consent forms. The surface markers of SHED were characterized using flow cytometry, while the differentiation potential was tested using osteogenic, adipogenic, and chondrogenic induction. For flow cytometry analysis, the cell surface expression levels of CD73, CD90, CD105, CD146, CD29, CD44, CD34, and CD45 were tested. Cells were washed twice with ice-cold PBS and stained with PE-or FITC-conjugated antibodies (Beyotime, Shanghai, China) for 30 min on ice in the darkroom. Samples were examined on an Accuri C6 flow cytometer (BD Biosciences, Franklin Lake, NJ, USA). FlowJo 7.6.1 analysis software was used to analyze the data. For three lineage inductions of SHED, an osteogenesis differentiation kit (Cyagen, Shanghai, China) was used to induce osteogenic differentiation. SHED were cultured in osteogenic differentiation medium and fed every three days for three weeks. Alizarin red working solution was used to stain the cells for 5 min. Adipogenesis was induced by using an adipogenic differentiation kit (Cellcook Biotech, Guangzhou, China). Oil Red O working solution (3:2 dilution with distilled water, filtered) was used to stain SHED cultured in adipogenic differentiation medium for 30 min. Chondrogenic induction of SHED was performed according to the instruction manual of the chondrogenic differentiation kit (Sanyuan, Shanghai, China). Alcian blue staining was applied after induction for 3–4 weeks.

### Measurement of cytokine and growth factor levels from serum-free cultured medium of SHED

SHED at 70–80% confluency were washed with PBS, and the culture medium was replaced with serum-free DMEM. After a 48 h incubation, the medium was collected and centrifuged for 10 min at 1000 × g. Matrix metalloproteinase-10 (MMP-10), vascular endothelial growth factor (VEGF), hepatocyte growth factor (HGF), and interleukin 6 (IL-6) in CM were determined by ELISA kits (Cloud-Clone, Guangzhou, China).

### Animal model establishment and cell transplantation

Eighty male SD rats (4–5 weeks old) were purchased from Hunan SJA Laboratory Animals. These rats were fed in the Laboratory Animal Research Center, Institutes for Life Sciences, South China University of Technology. All animal protocols were approved by the Research Ethics Committee of Guangdong Provincial People’s Hospital, Guangdong Academy of Medical Sciences (Approval ID: GDREC2019347A).

**Fig. 1:**
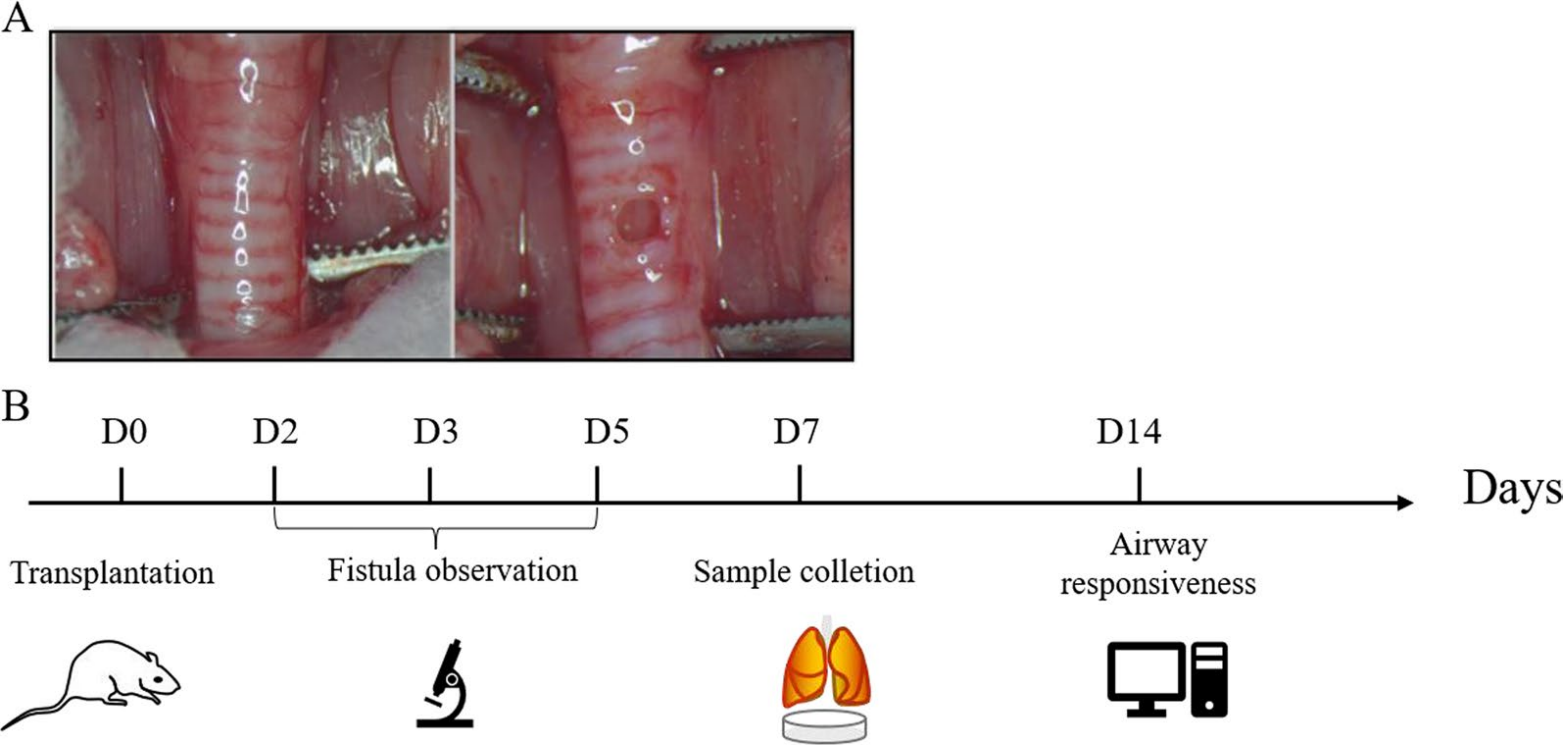
Establishment of the tracheal fistula and experimental flow. **A** Establishment of the tracheal fistula. After exposing the trachea, a 1.5-mm fistula was created on the trachea of SD rats with a biopsy punch. Under the microscope, each tracheal fistula had a uniform diameter and smooth edge. **B** Schematic illustration of the experimental flow. On D0, TF rats were transplanted with SHED around the fistula and via the tail vein, respectively. Fistula repair was observed on D2, D3, and D5. The tissues were collected on D7 for immunohistochemical, immunofluorescence, and western blotting assays. Airway responsiveness was measured on D14

A total of 80 rats were randomized into five groups: a sham-operated group, a local PBS group (L-PBS), an intravenous PBS group (I-PBS), a local SHED treatment group (L-SHED), and an intravenous SHED treatment group (I-SHED). To anesthetize the rats, 1% pentobarbital sodium (30 mg/kg) was given intraperitoneally. After a cervical incision was made, the skin, subcutaneous tissue, and muscle layers were dissected to expose the trachea. Then, a 1.5-mm fistula was created on the trachea of SD rats with a biopsy punch. Under the microscope, the fistula had a smooth edge and was uniform in diameter (Fig. 1A).

At the same time of the successful construction of the tracheal fistula, the L-SHED group was given a local injection of 1*10^7^ SHED in 500 µl of PBS, which was distributed at four sites around the fistula. The I-SHED group was given an intravenous injection of 1*10^7^ SHED in 1 ml of PBS. In the L-PBS and I-PBS groups, the rats received topical or intravenous application of an equal volume of PBS, respectively. On the 2nd, 3rd, and 5th days after the initial injection, the repair of the fistula was observed by a stereomicroscope. The fistula and lung tissues from these rats were harvested and processed for histological and immunostaining or western blotting one week after the injection. Airway responsiveness tests were performed two weeks after the injection (Fig. 1B).

### Histology and immunohistochemistry

The right lungs and fistulas were fixed in 4% paraformaldehyde (Sigma, St. Louis, USA) for 24 h. Tissues were then paraffin-embedded (Sigma, St. Louis, USA) and cut into 5-μm slices. Sections were stained with hematoxylin and eosin (H&E) (Sigma, St. Louis, USA) to assess the status of inflammatory infiltration, edema, and epithelial damage. Besides, Masson trichromatic dyeing solution was used to investigate connective tissue and collagen fibers. For immunohistochemical staining, lung tissues were incubated overnight at 4 °C with a rabbit polyclonal anti-Toll-like receptor 4 (TLR4) antibody (CST; 1:100), anti-interleukin (IL)-1β antibody (Cloud-Clone; 1:100), anti-IL-33 antibody (Cloud-Clone; 1:100), and anti-IL-4 antibody (Cloud-Clone; 1:100). Then, the slices were washed with PBS and incubated with goat anti-rabbit IgG and a streptavidin peroxidase (SP) complex for 40 min at 37 °C (ZSGB-BIO, Beijing, China). Immunoreaction was visualized after incubating with 0.05% diaminobenzidine and 0.003% H_2_O_2_. Finally, images were randomly taken using a digital pathology scanning system (Leica, Shanghai, China). The Smith score was used for semiquantitative analysis of pulmonary edema, alveolar and interstitial inflammation, alveolar and interstitial hemorrhage, and atelectasis and hyaline membrane formation. The average score of the two researchers was used to calculate the total lung injury score. Image-Pro Plus 6.0 was used as a tool to quantify the integrated optical density (IOD) of positive areas.

### Airway responsiveness of rats

On the 14th day, airway responsiveness to methacholine was measured in each group of three rats. After the rats were placed in the whole-body chamber and connected to the device (BUXCO, DSI, MN, USA), the basic Penh value was measured first. The respiratory data were collected with aerosols containing increasing doses of methacholine (6.25, 12.5, 25, and 50 mg/mL, with a volume of 0.5 mL) and analyzed with Flexi Vent software. Enhanced pause (Penh) was obtained as an index relative to the measured airway responsiveness.

### Immunofluorescence

Sections of the lungs were incubated in PBS containing 5% normal donkey serum and mouse antihuman nuclei (HuNu) antibody (Millipore; 1:200) overnight at 4 °C. After washing with PBS, the sections were incubated for one hour in a box at 37 °C with Alexa Fluor® 594 goat anti-mouse IgG (Invitrogen; 1:200). Then, the sections were redyed with DAPI, washed, and sealed.

### Western blotting

Total protein was extracted from the lung tissue in RIPA lysis buffer on ice with protease inhibitor cocktail. The concentration of total protein was determined using a BCA Protein Assay Kit. Equal amounts of proteins (20 μg) were separated by 10% sodium dodecyl sulfate–polyacrylamide gel electrophoresis (SDS-PAGE) before being transferred to a polyvinylidene difluoride (PVDF) membrane (Millipore, Billerica, MA, USA). The membranes were incubated overnight at 4 °C with specific primary antibodies against high mobility group box-1 (HMGB1) (CST; 1:1000), TLR4 (CST; 1:1000), and myeloid differentiation factor 88 (Myd88) (CST; 1:1000) after blocking in 5% milk/Tris-buffered saline Tween (TBST) for 2 h, followed by horseradish peroxidase (HRP)-labeled secondary antibodies for 2 h at room temperature. A ChemiScope series (Clinx, Shanghai, China) was used to observe immunoreactive bands, and Image-Pro Plus 6.0 was used to quantify the expression.

### Statistical analysis

GraphPad Prism 7.0 was used to analyze the data. All data are shown as the mean ± SEM. For the data statistics of the two experimental results, a T-test was applied to calculate the variance. For the data statistics of the five groups, an ANOVA with Turkey’s post hoc test was applied to obtain the variance between the groups. A value of *P* < 0.05 was considered statistically significant.

## Results

### Identification of SHED

We first examined the cell morphology, surface markers, and differentiation capability of SHED. Cell morphology was evaluated by light microscopy. The surface markers of SHED were detected using flow cytometry, while the differentiation potential was tested using osteogenic, adipogenic, and chondrogenic induction.

**Fig. 2:**
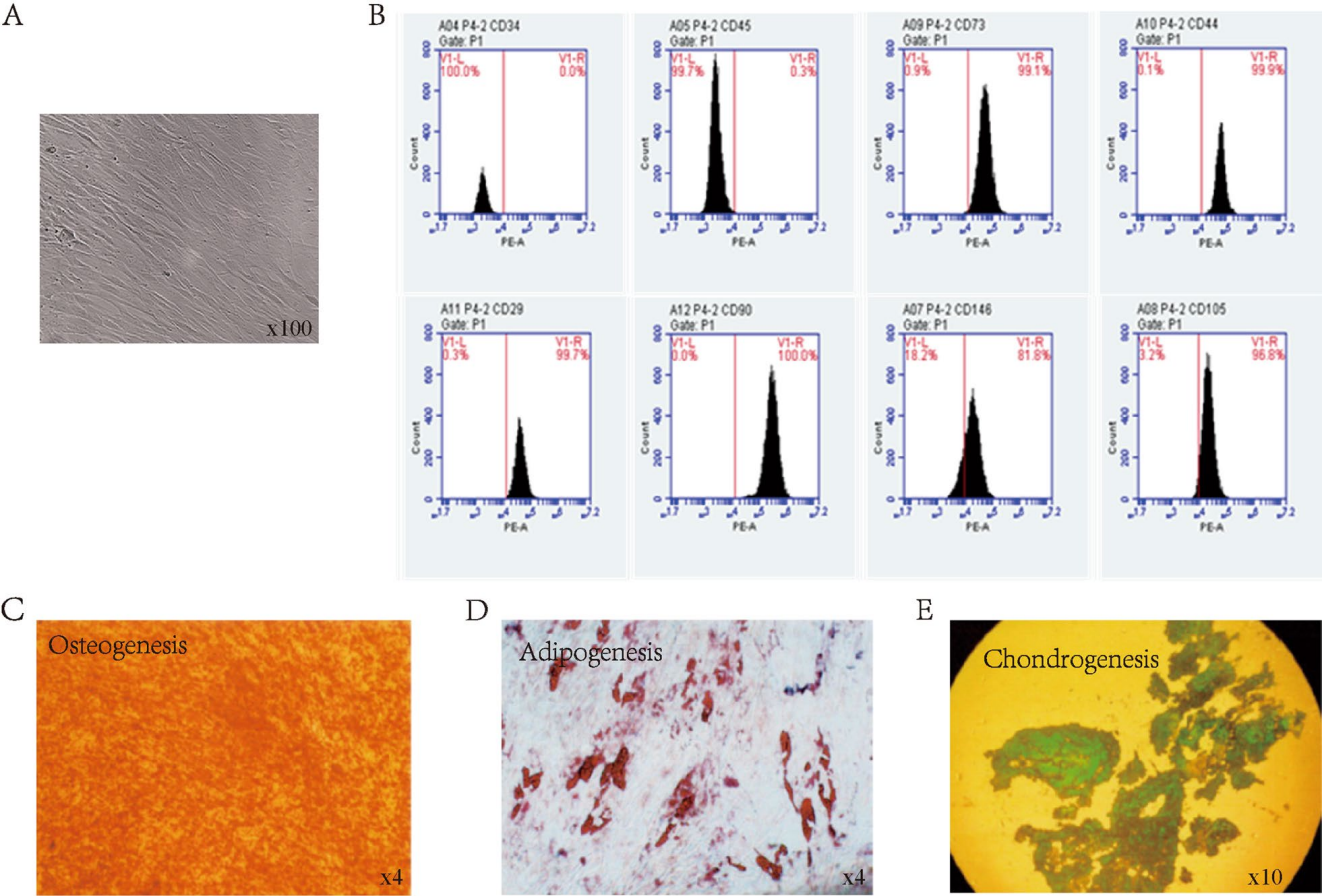
Identification and characterization of SHED. **A** Morphology of SHED. SHED grew adherently, with a long spindle form, an oval nucleus, and a fibroblast-like nucleus in the cytoplasm’s center. **B** Surface markers of SHED. Flow cytometric analysis showed that SHED highly expressed CD73, CD90, CD105, CD146, CD29, and CD44, while it did not express CD34 and CD45. **C** Osteogenic of SHED. Alizarin red staining of SHED. **D** Adipogenesis of SHED. Oil red O staining of SHED was performed after adipogenic induction. **E** Chondrogenesis of SHED. Alcian staining of SHED was applied after chondrogenic induction

Under the microscope, SHED adhered to the dish and grew in a long spindle shape (Fig. 2A). SHED were defined as having high levels of CD73, CD90, CD29, CD44, CD146, and CD105 expression while having low levels of CD34 and CD45 expression [23]. According to our flow cytometry results, the positive rates of CD73, CD90, CD29, and CD44 were higher than 95%, CD105 was higher than 90%, and CD146 was higher than 60%, while the positive rates of CD34 and CD45 were less than 2% (Fig. 2B). Differentiation of the SHED to osteocytes was confirmed by positive staining with alizarin red staining (Fig. 2C). After oil red O staining, deep red lipid droplets were visible, indicating adipogenic differentiation (Fig. 2D). Alcian staining was used to confirm chondrogenic differentiation (Fig. 2E). The three lineage inductions of SHED proved their strong differentiation potential. Many investigations have shown that MSC’s therapeutic efficacy is due to paracrine rather than cell differentiation. As a result, we discovered that SHED released several cytokines and growth factors, such as MMP-10, VEGF, HGF, and IL-6 (Table 1).

**Table 1:** Cytokine and growth factor levels are present in conditioned medium derived from SHED

Cytokine	Conditioned medium (n = 5, pg/ml)
MMP-10	300.23 ± 16.32
HGF	20.42 ± 3.88
VEGF	15.02 ± 2.47
IL-6	1.03 ± 0.09

The levels of MMP-10, HGF, VEGF, IL-6 in SHED were measured using ELISA kits (Cloud-Clone, China). Data are expressed as the mean ± standard deviation. HGF, hepatocyte growth factor. VEGF, vascular endothelial growth factor. IL-6: interleukin-6. MMP-10: matrix metalloproteinase-10

### Healing of fistulas after SHED transplantation

**Fig. 3:**
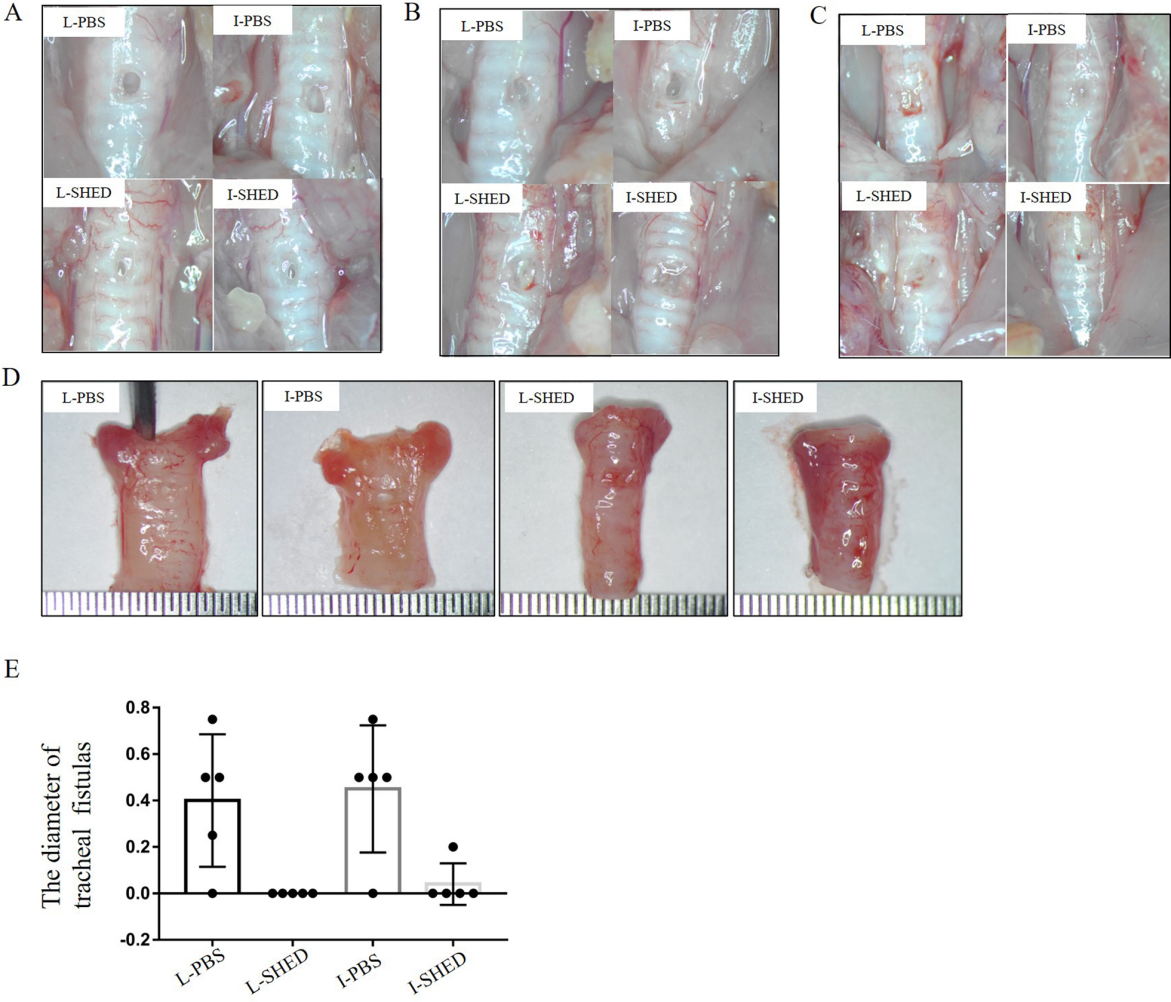
Healing of fistulas after SHED transplantation. **A**, **B**, **C** Healing of the fistulas on D2, D3, and D5. Fistula repair was observed under a stereomicroscope. The healing speed of the fistulas in the SHED-transplanted groups was faster than that in the PBS groups. **D** Representative gross images of isolated tracheal fistulas on D3. The diameters of the tracheal fistulas were 0.5 mm, 0.75 mm, 0 mm, and 0 mm. **E** Statistical analysis of the diameters of the isolated fistulas on day 3 (n = 5/group; *P < 0.05, compared with T test)

To investigate whether SHED had therapeutic effects on tracheal fistulas, we observed the healing of fistulas on the 2nd, 3rd, and 5th days after transplantation under a stereomicroscope. Compared to the respective PBS group, the fistulas in the SHED-transplanted groups healed substantially faster when observed under a stereomicroscope (Fig. 3A–C). Isolated tracheal fistulas were taken on the third day, and the diameters of the fistulas in each group were measured (Fig. 3D). The diameters of fistulas were significantly smaller in the SHED-transplanted groups than in the PBS groups (Fig. 3E). These findings imply that SHED transplantation, whether in the form of a topical multipoint injection around the fistula or an intravenous SHED injection via the tail vein, could accelerate fistula healing in rats.

### Engraftment of SHED in the lung and fistula

**Fig. 4:**
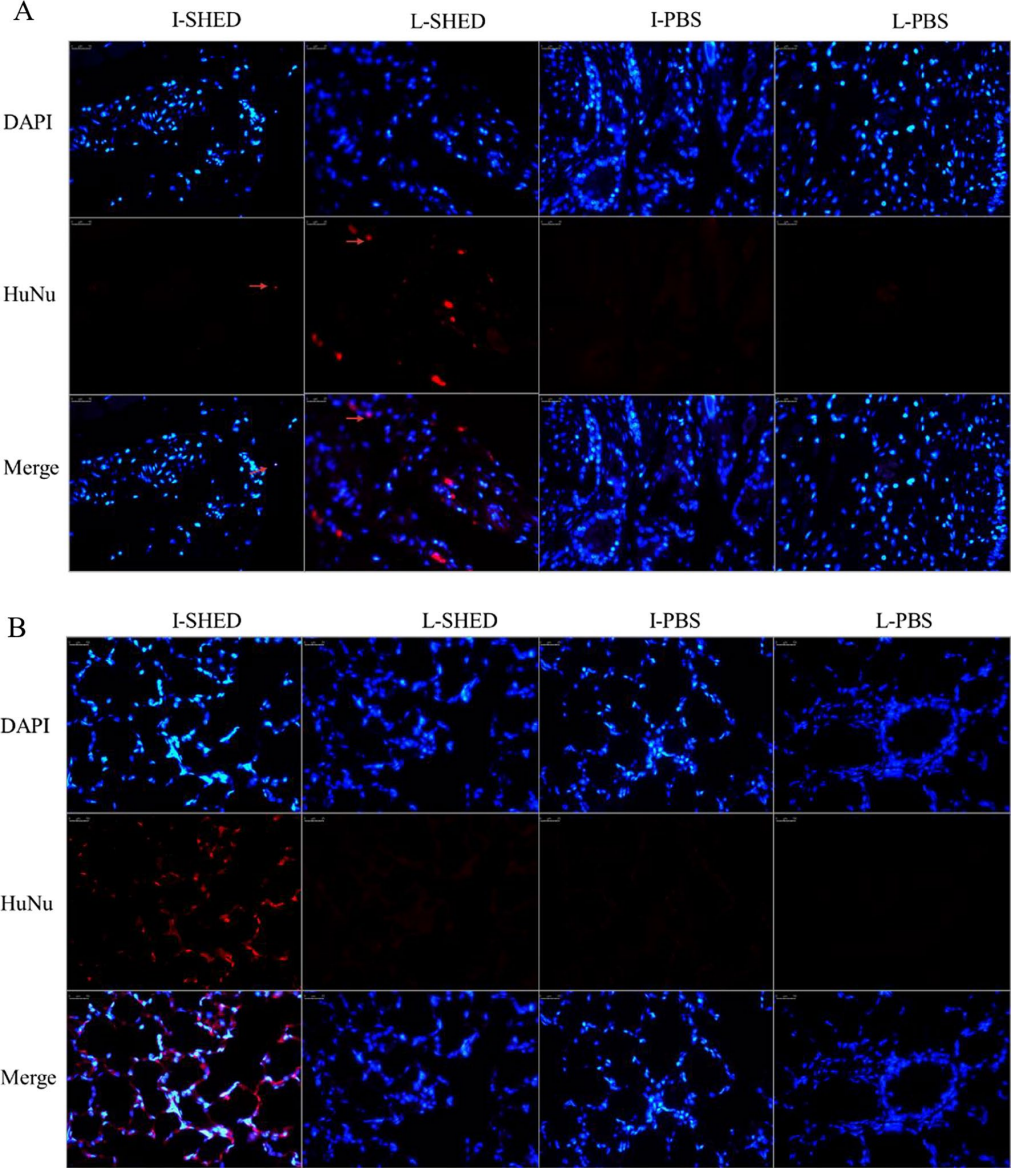
Engraftment of SHED around the fistula and in the lung. **A** Surviving SHED after transplantation around the fistula. SHED were found in both the I-SHED group and the L-SHED group. No SHED were observed in the I-PBS and L-PBS groups. **B** Surviving SHED after transplantation in the lung. SHED were found in the I-SHED group. However, no transplanted SHED were seen in the other groups

To determine whether SHED engrafted to the injury site in vivo, the fistula and lung tissues were harvested and cut into 5-µm-thick slices on the 7th day. The tissues were next stained with a mouse antihuman nuclei (HuNu) antibody, a human-specific nucleus marker that recognizes human and primate nuclei but not rat or mouse nuclei. After intravenous injection, SHED was engrafted into the lungs and the fistulas (Fig. 4A, B). However, when locally injected, SHED were found only around fistulas but not in the lungs. As expected, no signal was detected in the lungs or fistulas in the L-PBS and I-PBS groups. These results suggest that SHED in the body entered circulation and migrated to the wounded area.

### Pathology of the fistulas

**Fig. 5:**
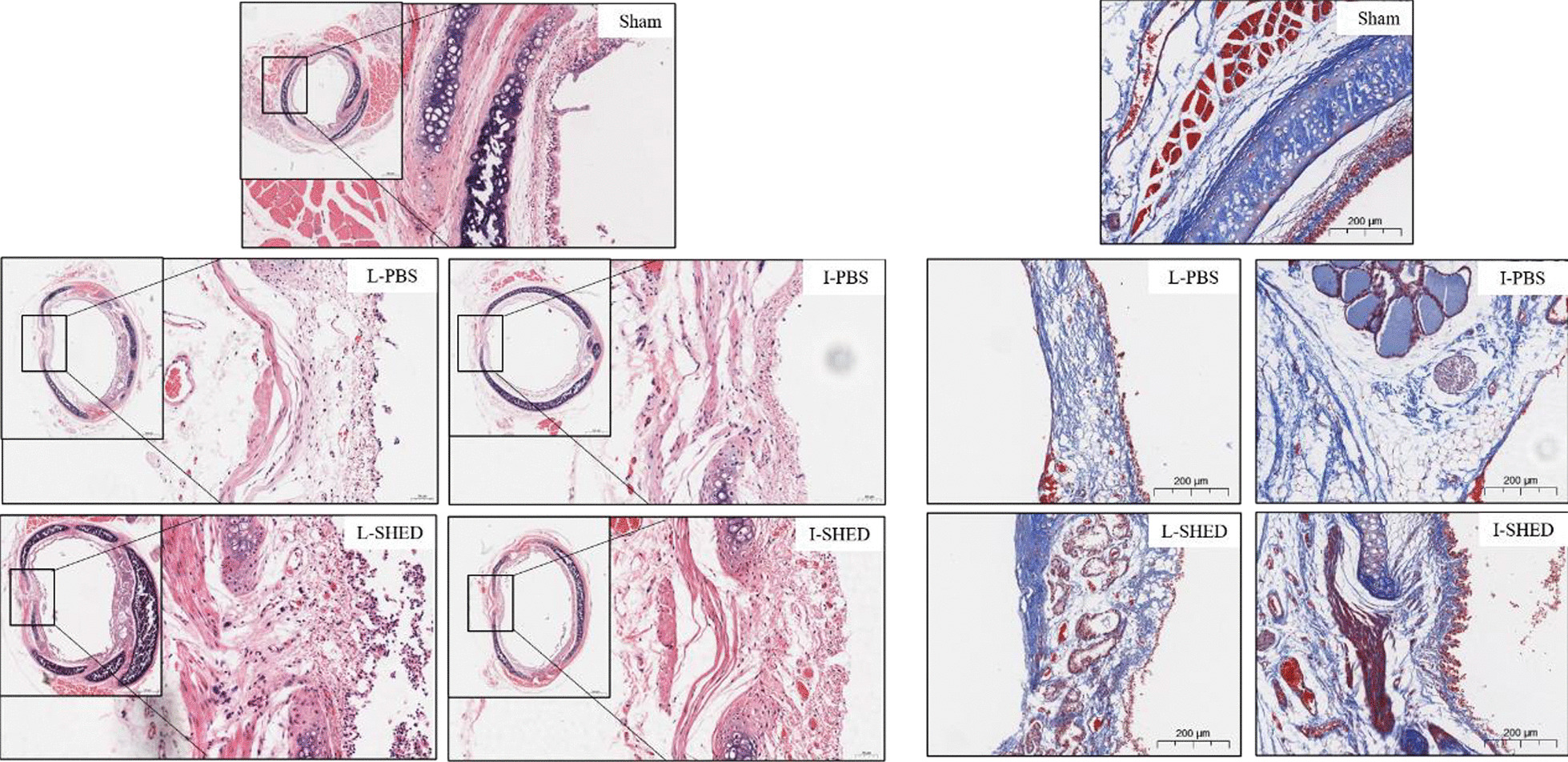
H&E and Masson staining of the fistula. Fistula tissues in the SHED treatment groups showed obvious inflammatory cells and intense neovascularization, and the hyperplasia of fibers and epithelium

The tracheal fistula tissues of rats in each group were taken on day 7, and H&E and Masson staining analyzed the pathological patterns of the tissues around the fistulas. When compared with the PBS groups, fistula tissues in the SHED treatment groups showed apparent inflammatory cell infiltration, extensive neovascularization formation, and hyperplasia of fibers and epithelium, indicating that SHED could have the potential to be conducive to tissue repair (Fig. 5). But in both the I-SHED and L-SHED treatment groups, the fistulas demonstrated incomplete tissue repair and healing.

### Lung inflammation and airway responsiveness

**Fig. 6:**
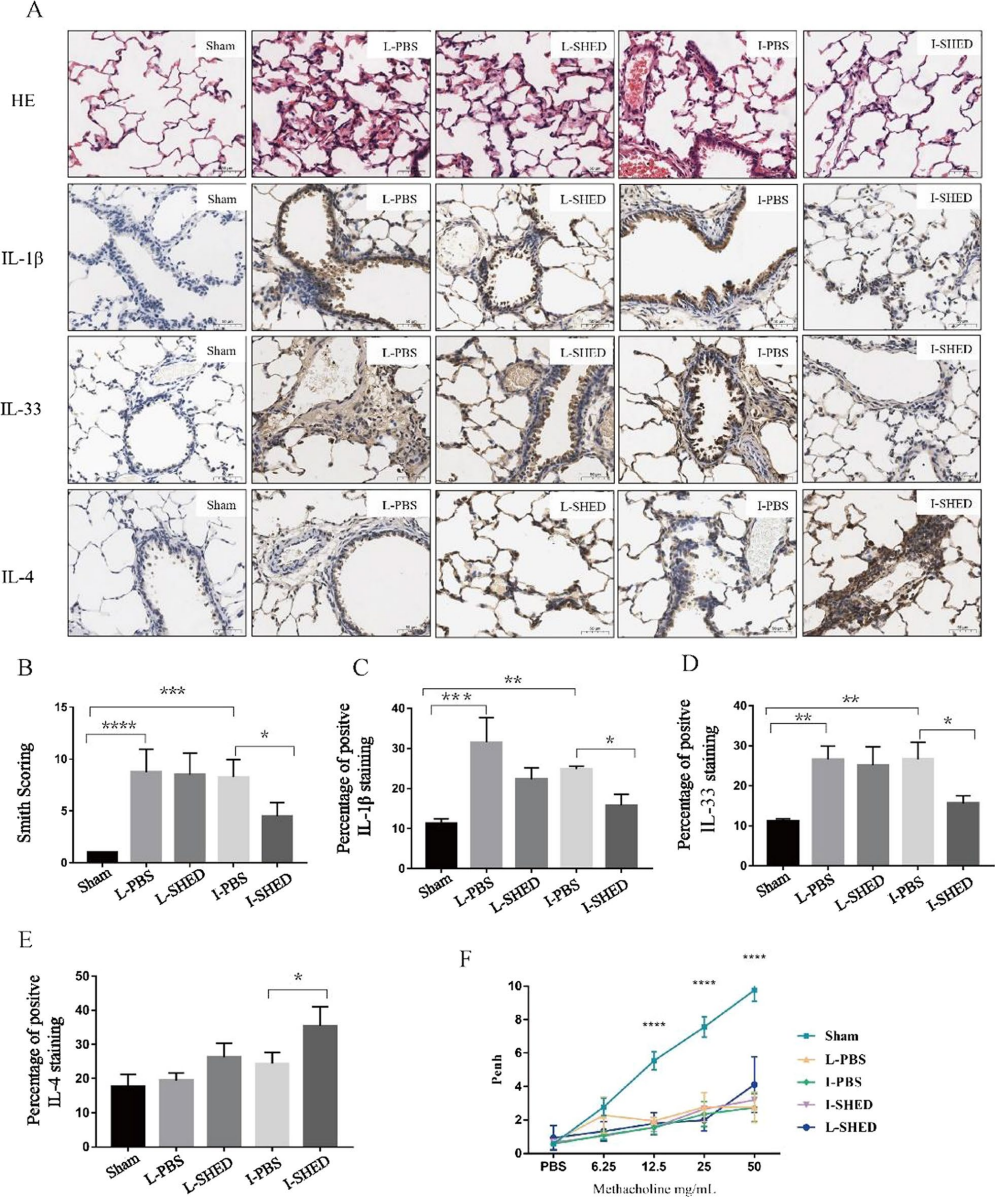
Lung inflammation and Airway responsiveness. **A** HE and Immunohistochemical staining of lung tissue sections using IL-1β, IL-33, and IL-4. **B** The compassion in Smith scoring of H&E staining. **C-E** Quantification of IL-1β, IL-33, and IL-4 staining in the lungs. **F** Airway responsiveness of rats. (n = 3/group;**P < 0.01, ***P < 0.001, and ****P < 0.0001 compared with one-way ANOVA with Turkey’s post hoc tests)

H&E staining and smith scoring of lung tissue showed intravenous SHED treatment could induce damage to the lung tissues caused by TF, whereas local treatment could not (Fig. 6A, B). To further assess the status of the inflammatory level in the lung tissues, we identified several pro-inflammatory and anti-inflammatory factors by immunohistochemical staining. The expressions of IL-1β and IL-33 were decreased, while IL-4 was elevated in the I-SHED group compared with the I-PBS group (Fig. 6A–E). These results suggest that intravenous SHED injections could successfully reduce lung inflammation, whereas local SHED injections did not.

Airway responsiveness was assessed using whole-body plethysmography on day 14. The enhanced pause (Penh) test was used to determine airway responsiveness. As the concentration of methacholine increased, the rats in each group showed much deeper, rapider, and open-mouth breathing. Furthermore, the Penh values curve elevated slowly and flatly in the SHED or PBS treatment groups, while the Penh values curve in the sham-operated group expressed a bigger and relatively significant increase. The Penh values of the sham-operated group were significantly higher than those in the SHED or PBS treatment groups (*P* < 0.001) when the concentration of methacholine reached above 12.5 mg/ml. There was no significant difference between the I-SHED or L-SHED groups and their respective PBS groups (Fig. 6F).

### The expression of TLR4 in the lungs after transplantation

**Fig. 7:**
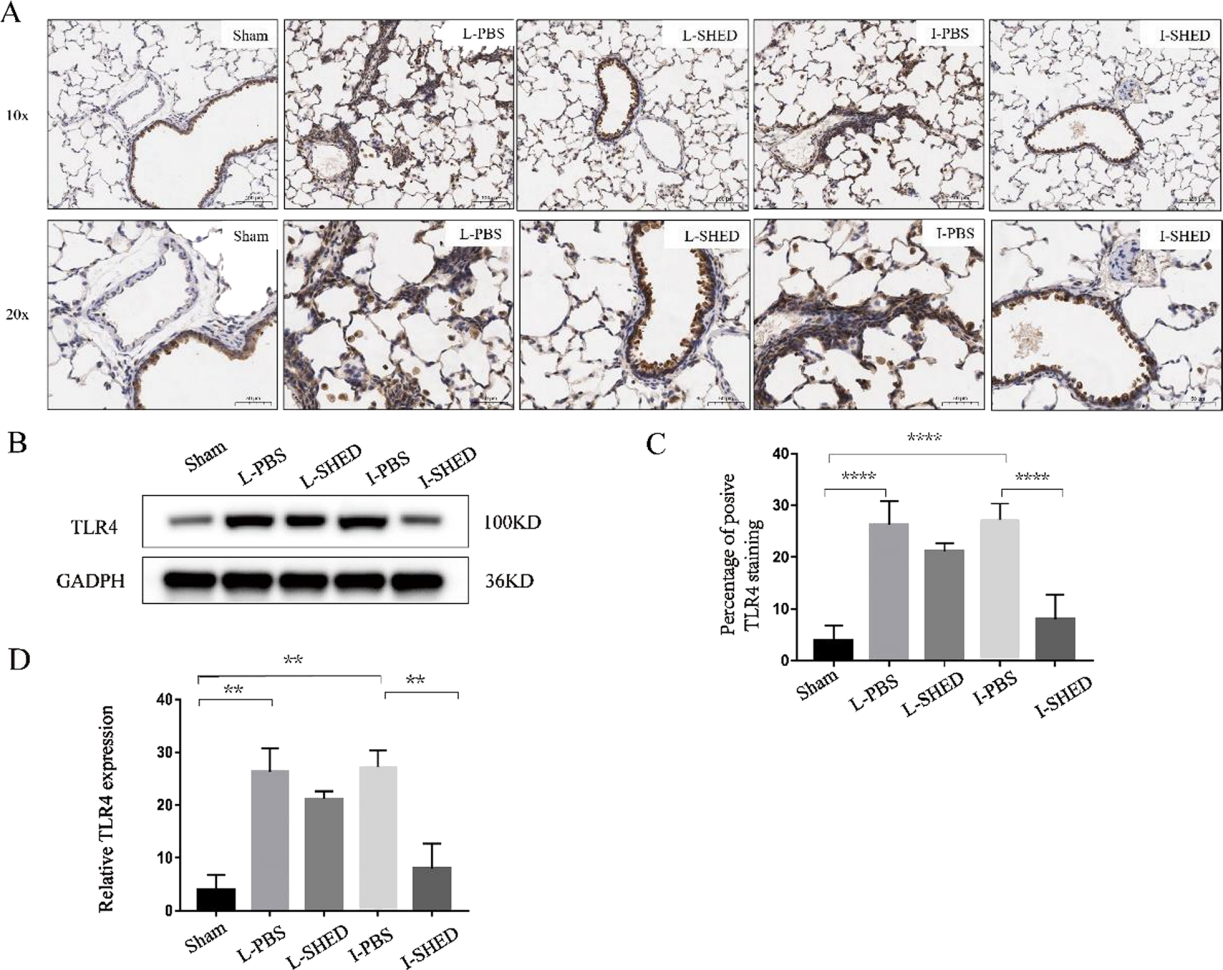
TLR4 expression levels in the lungs using immunohistochemistry and western blotting. **A** Immunohistochemical staining of TLR4 in the lungs. **B** Western blotting of TLR4. **C** Quantification of TLR4 staining in the lungs. **D** Relative expression of TLR4 standardized to GAPDH. (n = 3/group; **P < 0.01, ***P < 0.001, and ****P < 0.0001 compared by one-way ANOVA with Turkey’s post hoc tests)

To further explore the molecular changes after SHED transplantation in TF rats, immunohistochemical staining of TLR4 was performed in the lung tissue. Toll-like receptors are a class of crucial transmembrane protein molecules that can recognize foreign microbial molecules or pathogen-associated molecular patterns and participate in nonspecific immunoreactions. Stem cells have previously been shown to affect the function of natural killer cells, macrophages, and T lymphocytes via a TLR-dependent response [24]. TLR4 expressed on stem cells serves as a crucial sensor that stimulates LPS-induced cell signaling via myeloid differentiation factor 88 (MyD88) to downstream proinflammatory genes, ultimately defining the adaptive stem cell response [25]. Liu found that MSC exhibited its anti-inflammatory effects by inhibiting activation of the TLR4/NF-κB signaling pathway [26]. In our study, the expression of TLR4 was significantly increased in the L-PBS and I-PBS groups compared with the sham-operated group by immunohistochemical staining (Fig. 7A, C). After intravenous injection of SHED, the expression levels of TLR4 (*P* < 0.0001) in the lung were significantly reduced when compared to the I-PBS group. After topical SHED treatment, the expression levels of TLR4 in the lung showed a downward trend, but there was no statistical significance when compared to the L-PBS group. Consistent results were obtained by western blot analysis (Fig. 7B, D).

### The HMGB1/TLR4/MYD88 signaling pathway reactivity

**Fig. 8:**
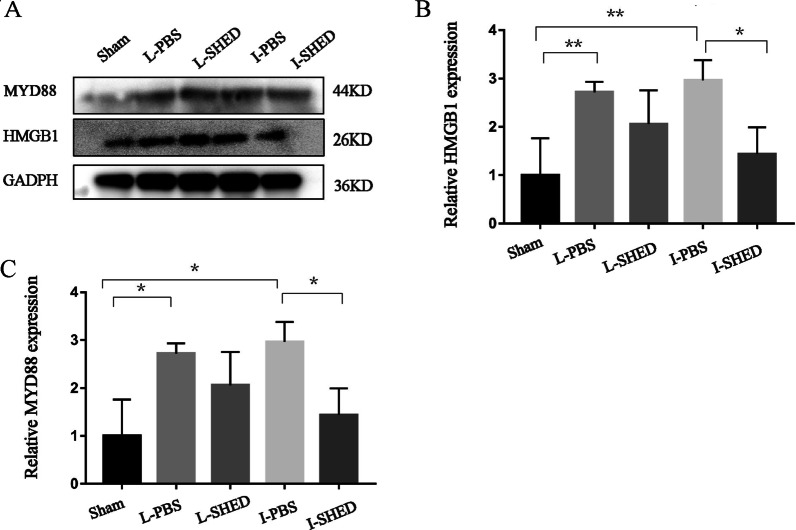
HMGB1 and MYD88 expression levels in the lungs using western blotting. **A** Western blotting of HMGB1 and MYD88. **B** Relative expression of HMGB1 standardized to GAPDH. **C** Relative expression of MYD88 standardized to GAPDH. (n = 3/group; *P < 0.05, **P < 0.01, and ***P < 0.001 compared by one-way ANOVA with Turkey’s post hoc tests)

The preceding results indicated that the expression and activation of TLR4 were decreased after intravenous SHED transplantation. It has been reported that high mobility group box 1 (HMGB1) binds to TLR4, leading to MYD88-dependent activation of the NF-KB pathway in neutrophils and macrophages and promoting inflammatory responses [27]. We examined the expression levels of HMGB1 and MyD88 using western blotting (Fig. 8A). In the L-PBS and I-PBS groups, the HMGB1 and MYD88 expression levels were significantly increased compared with the sham-operated group (Fig. 8B, C). After intravenous injection of SHED, the expression of HMGB1 and MYD88 significantly decreased in the I-SHED group compared with the I-PBS group. However, there was no statistical significance between the L-SHED group and the L-PBS group.

## Discussion

Tracheal fistulas (TF) can be dangerous and even fatal. Currently, there is no practical way to treat it. Therefore, there is an urgent need to find novel therapeutic interventions. To our knowledge, the present study was the first to establish the TF rat models to determine the effect of SHED treatment. First, we identified SHED. SHED grew in fusiform adherents, expressing the specific molecules CD73, CD90, CD29, CD44, CD105, and CD146, and demonstrated the ability to differentiate into osteogenic, lipogenic, and chondrogenic cells. It suggests that our SHED has the characteristics of stem cells consistent with the previous research, so that it could be used for further research. Second, an animal model of respiratory tract fistula was established. In our study, we constructed a tracheal fistula model in rats using a 1.5 mm surgical punch. Under the microscope, the fistula had a smooth edge and was consistent in diameter. In 2000, Wagner et.al [28] proposed the surgical construction of a minipig model of tracheoesophageal fistulas. In addition, left pneumonectomy was performed to establish a bronchopleural fistula model in Wistar rats [29]. However, the surgery caused severe harm to the animals, and controlling the diameter and margin of the fistula was challenging. Then, Gao et al. [30] used a magnetic compression approach to create dog models with a tracheoesophageal fistula. The magnetic compression technique solved the defects of fistulas with different diameters and uneven edges. However, it took a long time for the fistulas to develop and required an X-ray examination. To our knowledge, this is the first study that reported a rat model of tracheal fistula. Our approach has the advantages of being easy to operate and fast to develop. Moreover, the fistulas had consistent diameters and smooth margins, indicating that this approach could be utilized as an animal model to research respiratory fistulas.

Next, we observed the therapeutic effect of SHED on tracheal fistulas. The findings revealed that SHED transplantation could accelerate the rate of fistula healing in rats. Currently, there are few animal experiments on stem cell therapy for respiratory tract fistulas, and most of them are clinical case reports [31–33]. Petrella et al. [10] conducted a study on the efficacy and safety of BMSC injections under bronchoscopy in the treatment of a BPF goat model, and the results showed that BMSCs could repair and cure BPF. SHED are defined as one source of MSCs that have great potential in tissue repair [34]. In our study, the diameters of fistulas in the SHED-transplanted groups were considerably lower than those in the PBS groups on the third day. And pathological analysis demonstrated the formation of new blood vessels surrounding the fistula and the proliferation of collagen fibers and epithelium in the SHED-treated groups. Inflammatory cells may play a pivotal role in wound healing at this stage of the cicatrization process. They assist in microbial decontamination, produce multiple growth factors that promote the healing process and help in the clearance of cellular death and tissue remodeling. On the 7th day after the MSC treatment of the bronchial stump model of Wistar rats, David et al. [29] found intense inflammation with diffuse ulceration of the mucosa, intense neovascularization, and signs of infection with foreign body granulomas in the peribronchial pleura, which was similar to our study. He suggested that MSCs administered topically on a bronchial stump could migrate and reach the bronchial wall and participate in the healing process.

We used two different methods to transplant SHED; topical treatment and intravenous treatment. Both of them have the potential to hasten fistula repair. Moreover, SHED may survive in the lungs after intravenous injection, and intravenous SHED could reduce pulmonary inflammation. It may be because intravenously injected MSCs can migrate to the lung through circulation and tend to recruit into the injury site. One hour after an injection of human MSCs from the jugular veins of rats, the number of cells decreased by 82%, predominantly in the lungs, liver, and kidneys, with a few in other organs, such as the kidneys and small intestine, and the number of cells decreased over time [35]. When pulmonary damage exists, the retention of stem cells increases. The local lung microenvironment activates MSC, releasing substantial amounts of anti-inflammatory substances that reduce inflammation. Even an intravenous injection of substance P can activate mesenchymal stem cells in the body, causing them to enter the bloodstream and travel to the injury site, facilitating tissue regeneration [36]. Our study suggests that both topical and intravenous therapies have the potential to speed up fistula repair. And intravenous treatment may be more effective in reducing pulmonary inflammation.

According to research on their dynamic dispersion in vivo, MSCs may exert their therapeutic benefits through touch-and-walk or hit-and-run processes [37]. Many studies have shown that the therapeutic efficacy of MSCs involves multiple targets and strategies rather than cell differentiation [38]. Previous studies have suggested that the paracrine effect of MSCs plays an essential role in trauma repair and regulation of the inflammation response. According to our findings, SHED released a variety of cytokines and growth factors, including MMP-10, VEGF, HGF, and IL-6. These elements may aid in the healing of fistulas and alleviate pulmonary inflammation.

Finally, we further explored the molecular mechanism by which SHED affects its effect on reducing lung inflammation. TLR4 is present in immune cells such as macrophages, neutrophils, and nonimmune cells, including alveolar endothelial cells and epithelial cells. Endothelial cell destruction is the basis of structural degradation of the alveolar basement membrane in lung injury, and infiltration of alveolar inflammatory cells is the key to the inflammatory response. Furthermore, earlier research has suggested that TLR4-mediated inflammation is involved in acute lung injury caused by lipopolysaccharide (LPS). These changes lead to the synthesis and release of various inflammatory mediators and ultimately initiate and amplify the inflammatory response. TLR4 disruption has been shown to reduce pulmonary inflammation and damage in previous studies [39]. MSCs could downregulate the expression of TLR4 to inhibit lung inflammation [40, 41]. MSCs were discovered to have an anti-inflammatory effect on renal fibrosis by suppressing the activation of the TLR4 signaling pathway [26]. MSC exosomes have also been shown to diminish burn-induced inflammation by inhibiting the TLR4 signaling pathway [42]. Our results also suggested that intravenous SHED treatment might reduce the inflammatory response mediated by the HMGB1/TLR4/MYD88 signaling pathway. We believe that regulating the effect of SHED is multifaceted, which is also a significant feature of biologically active drugs that are different from chemical single-component drugs. However, further investigations are still warranted.

There are some limitations to our current study. First of all, our infusion was a single injection, and a single dose of SHED had a short-term effect. Furthermore, since our study only used a single infusion of SHED, long-term tolerance to tissue allografts was unable to be achieved. As a result, immunological rejection was inescapable, speeding up SHED clearance in rats. Although our studies provide encouraging results that suggest that SHED treatment is a promising therapy for TF, safety remains one of the main concerns in cell therapy. To develop safe and effective stem cell-based treatment approaches, secure SHED must be produced, as well as quality control systems, delivery mechanisms, and strict adherence to the Clinical Translation of Stem Cells Guidelines.

## Conclusion

In summary, SHED could accelerate tracheal fistula healing and reduce lung inflammation. Furthermore, intravenous SHED injection inhibited the activation and expression of HMGB1/TLR4/MYD88 signaling pathways, demonstrating that SHED protects in a bystander-like manner. These findings suggest that SHED transplantation may be a potential solution to repair respiratory tract fistulas. Although our experimental results showed that SHED had a healing effect on tracheal fistulas, more in-depth investigations are needed to promote future clinical applications.

## Data Availability

The datasets used and analyzed during the current study are available from the corresponding author upon reasonable request.

## Abbreviations

SHED: Stem cells from human exfoliated deciduous teeth
TF: Tracheal fistulas
TLR4: Toll-like receptor 4
MSCs: Mesenchymal stem cells
HMGB1: High mobility group box 1
MYD88: Myeloid differentiation factor 88
IL-1β: Interleukin-1β
IL-33: Interleukin-33
IL-4: Interleukin-33
IL-6: Interleukin-6
VEGF: Vascular endothelial growth factor
HGF: Hepatocyte growth factor
MMP-10: Matrix metalloproteinase-10
