# Correction: The therapeutic effect of stem cells from human exfoliated deciduous teeth on a rat model of tracheal fistula

**DOI:** 10.1186/s13287-022-03195-2

**Published:** 2022-10-20

**Authors:** Fang Wang, Zhangwen Li, Feng-Juan Lyu, Jie Gao, Jinle Lin, Jianling Liu, Xiaowen Chen, Zhongpeng Li, Jiajie Shan, Jian Wu

**Affiliations:** 1 School of Medicine, South China University of Technology, Guangzhou, 510006 China; 2 Second Department of Elderly Respiratory, Guangdong Provincial People’s Hospital, Guangdong Academy of Medical Sciences, Guangdong Provincial Geriatrics Institute, Guangzhou, 510080 China; 3 The Second School of Clinical Medicine, Southern Medical University, Guangzhou, 510515 China; 4 Department of Emergency Medicine, People’s Hospital of Shenzhen Baoan District, The Second Affiliated Hospital of Shenzhen University, 518101 Shenzhen, China

## Correction to: Stem Cell Research & Therapy (2022) 13:310 10.1186/s13287-022-02994-x

Following the publication of the original article [1], the authors identified some errors.

1: The authors noticed that the figure legend of Fig. 4 should be added with positive HuNu staining (red) to provide additional clarification. The updated figure legend of Fig. 4 is provided in this correction.

2: Fig. 7D was mistakenly duplicated from 7C. The correct Fig. 7D is provided in this correction.

3: Fig. 8C was mistakenly duplicated from 8B. The correct Fig. 8C is provided in this correction.

4: The affiliation of the corresponding author is not accurate. The footnote on the first page should be changed to “^2^Second Department of Elderly Respiratory, Guangdong Provincial People’s Hospital, Guangdong Academy of Medical Sciences, Guangdong Provincial Geriatrics Institute, Guangzhou, 510,080, China.”

It has been corrected after the authors double checked the original data. The results and conclusions concluded in this paper are still valid.

**Legend for figure 4:**

**Fig. 4** Engraftment of SHED around the fistula and in the lung. **A** Surviving SHED (positive HuNu staining (red)) after transplantation around the fistula. SHED were found in both the I‑SHED group and the L‑SHED group. No SHED were observed in the I‑PBS and L‑PBS groups. **B** Surviving SHED (positive HuNu staining (red)) after transplantation in the lung. SHED were found in the I‑SHED group. However, no transplanted SHED were seen in the other groups.

**Fig. 7:**
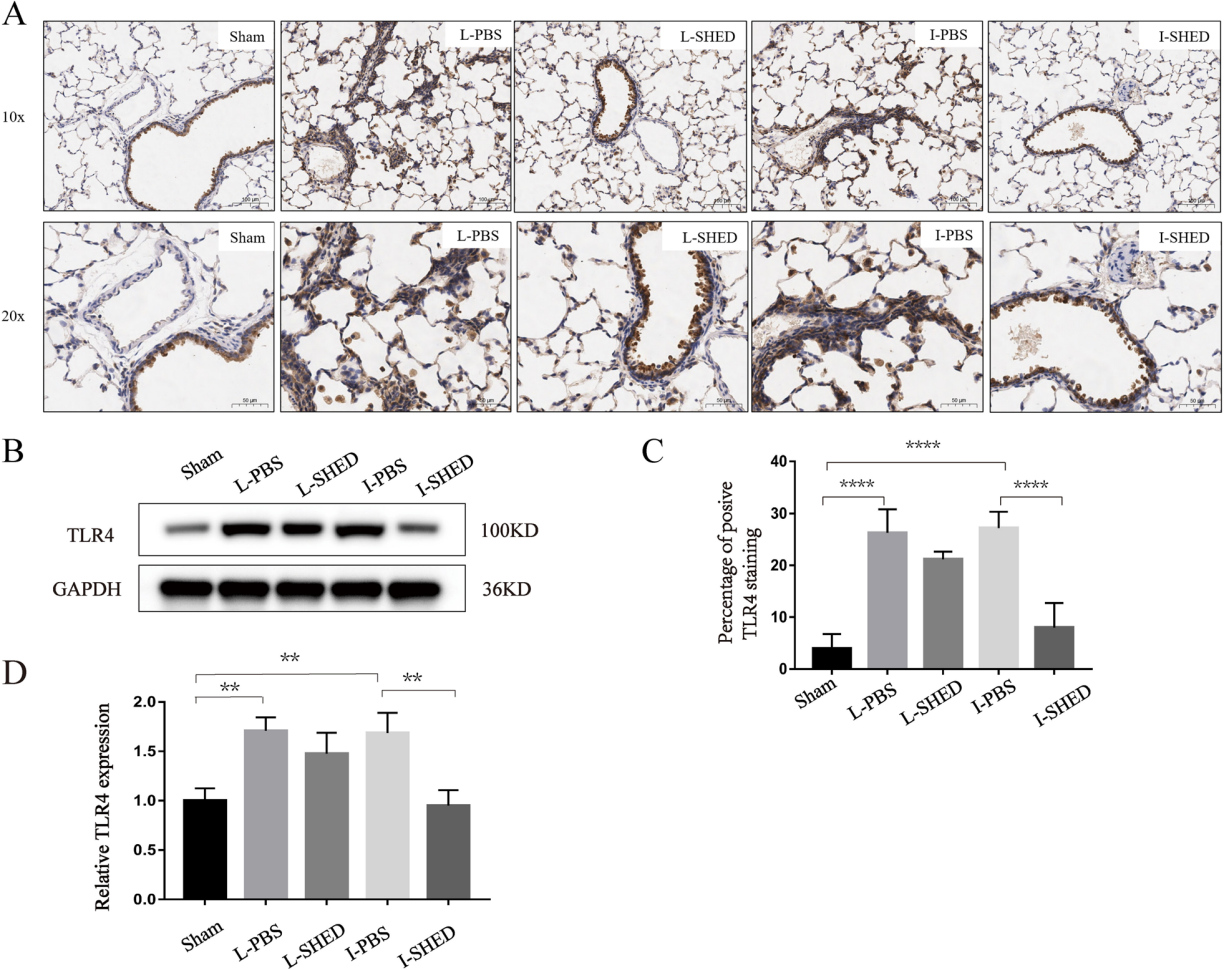
TLR4 expression levels in the lungs using immunohistochemistry and western blotting. **A** Immunohistochemical staining of TLR4 in the lungs. **B** Western blotting of TLR4. **C** Quantification of TLR4 staining in the lungs. **D** Relative expression of TLR4 standardized to GAPDH. (n = 3/group; *P < 0.05, **P < 0.01, and ***P < 0.001 compared by one-way ANOVA with Turkey’s post-hoc tests)

**Fig. 8:**
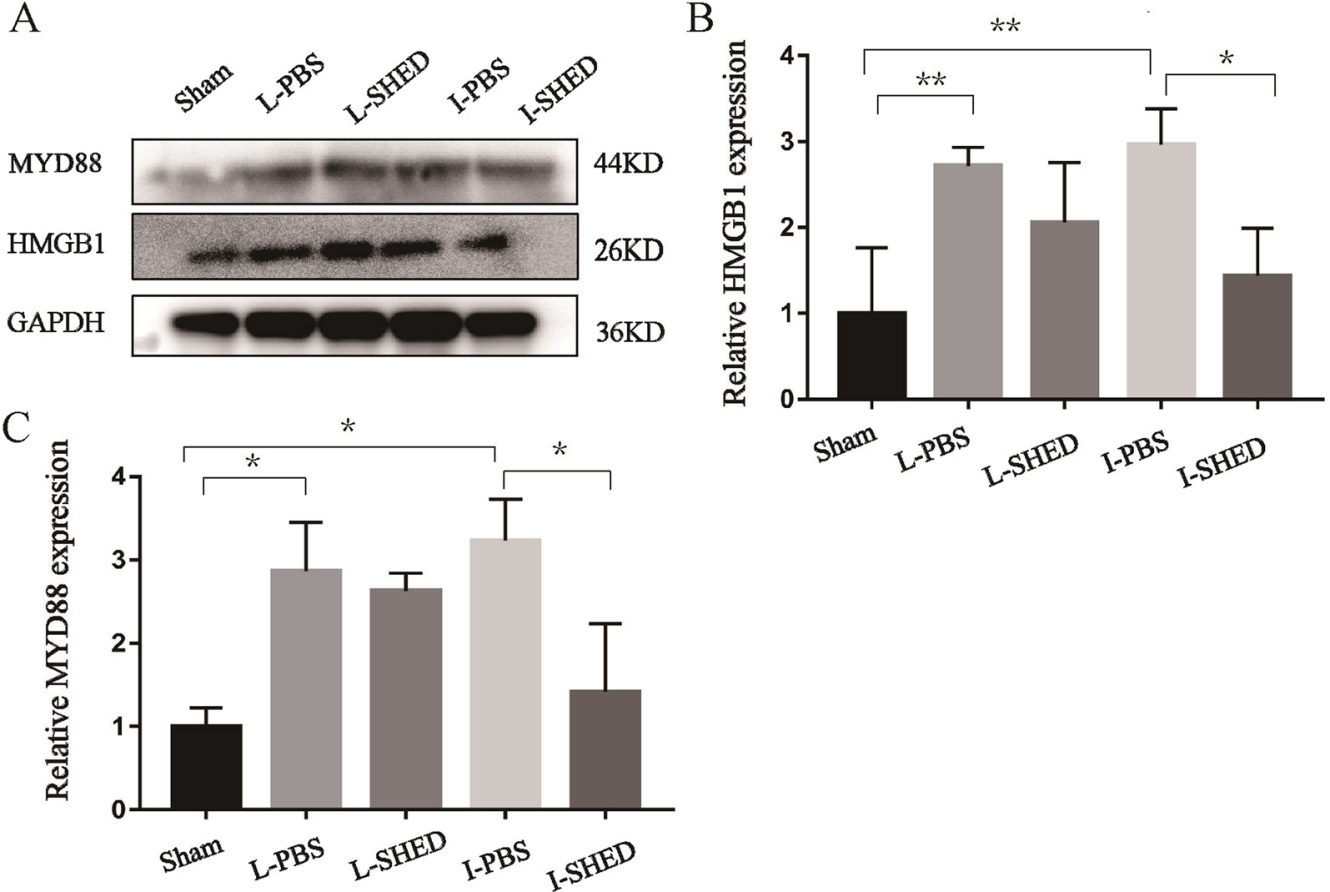
HMGB1 and MYD88 expression levels in the lungs using western blotting. **A** Western blotting of HMGB1 and MYD88. **B** Relative expression of HMGB1 standardized to GAPDH. **C** Relative expression of MYD88 standardized to GAPDH. (n = 3/group; *P < 0.05, **P < 0.01, and ***P < 0.001 compared by one-way ANOVA with Turkey’s post-hoc tests)
